# Effect of a program of short bouts of exercise on bone health in adolescents involved in different sports: the PRO-BONE study protocol

**DOI:** 10.1186/s12889-015-1633-5

**Published:** 2015-04-11

**Authors:** Dimitris Vlachopoulos, Alan R Barker, Craig A Williams, Karen M Knapp, Brad S Metcalf, Luis Gracia-Marco

**Affiliations:** Children’s Health and Exercise Research Centre. Sport and Health Sciences, University of Exeter, Exeter, UK; Growth, Exercise, Nutrition and Development Research Group, University of Zaragoza, Zaragoza, Spain; Department of Medical Imaging, College of Engineering, Mathematics and Physical Sciences, University of Exeter, Exeter, UK; University of Exeter Medical School, Exeter, UK

**Keywords:** Body composition, Longitudinal study, Plyometric jump training intervention, Osteogenic, Non-osteogenic, Sport participation, Weight-bearing exercise

## Abstract

**Background:**

Osteoporosis is a skeletal disease associated with high morbidity, mortality and increased economic costs. Early prevention during adolescence appears to be one of the most beneficial practices. Exercise is an effective approach for developing bone mass during puberty, but some sports may have a positive or negative impact on bone mass accrual. Plyometric jump training has been suggested as a type of exercise that can augment bone, but its effects on adolescent bone mass have not been rigorously assessed. The aims of the PRO-BONE study are to: 1) longitudinally assess bone health and its metabolism in adolescents engaged in osteogenic (football), non-osteogenic (cycling and swimming) sports and in a control group, and 2) examine the effect of a 9 month plyometric jump training programme on bone related outcomes in the sport groups.

**Methods/Design:**

This study will recruit 105 males aged 12–14 years who have participated in sport specific training for at least 3 hours per week during the last 3 years in the following sports groups: football (n = 30), cycling (n = 30) and swimming (n = 30). An age-matched control group (n = 15) that does not engage in these sports more than 3 hours per week will also be recruited. Participants will be measured on 5 occasions: 1) at baseline; 2) after 12 months of sport specific training where each sport group will be randomly allocated into two sub-groups: intervention group (sport + plyometric jump training) and sport group (sport only); 3) exactly after the 9 months of intervention; 4) 6 months following the intervention; 5) 12 months following the intervention. Body composition (dual energy X-ray absorptiometry, air displacement plethysmography and bioelectrical impedance), bone stiffness index (ultrasounds), physical activity (accelerometers), diet (24 h recall questionnaire), pubertal maturation (Tanner stage), physical fitness (cardiorespiratory and muscular), bone turnover markers and vitamin D will be measured at each visit.

**Discussion:**

The PRO-BONE study is designed to investigate the impact of osteogenic and non-osteogenic sports on bone development in adolescent males during puberty, and how a plyometric jump training programme is associated with body composition parameters.

## Background

Osteoporosis is a common skeletal disease associated with high morbidity and mortality [1]. Approximately 2.7 million European men and women suffer an osteoporotic fracture every year [2]. The economic burden of osteoporosis in Europe is higher than most types of cancer (except lung cancer), or chronic cardiorespiratory diseases [2,3] and represents a direct annual cost of ~ €31.7 billion to health care and social services [1]. In order to improve the outcome for osteoporosis, primary prevention remains the most important policy action in public health. Although contested [4], it is generally accepted that acquiring a high bone mass during childhood and adolescence is a key determinant of adult skeletal health [5-7]. Approximately 60% of osteoporotic cases in adult life are related to low bone mineral content (BMC) in adolescence with up to 50% of total body (TB) bone mass achieved during this period of life [8,9]. Peak bone mass attainment typically occurs between the second and third decade of life, with 80-90% acquired by late adolescence, although this is skeletal site dependent [6,10]. Although bone mass is ~ 60-80% genetically determined [11], there are other factors strongly related with bone mass development. Environmental and lifestyle factors such as physical activity (PA) [12] and nutrition, i.e. calcium intake [13] and vitamin D [14], are known to have important osteogenic effects and have been the key focus in several interventions.

### Exercise as a tool to improve bone health

Exercise has been proposed as a key factor for developing healthy bones in childhood and adolescence [15,16], mainly when high-impact and weight-bearing PA occurs [15] above a certain intensity and duration [15,17,18]. Longitudinal studies have shown that habitual PA is positively associated with bone health in children and adolescents because of its impact on bone development [19,20]. The long-term positive effects of PA during adolescence remain into young adulthood with active males aged 24.2 years having 8 and 10% higher BMC at TB and femoral neck (FN) respectively compared to non-active peers, even when adjusted for maturation and size [21]. Research conducted on former professional football players showed that exercise is not only an important factor in the accretion of, but also in the maintenance, of bone mineral density (BMD) [22]. It has been shown that moderate and readily accessible weight-bearing exercise before puberty may increase femoral volumetric BMC, by increasing cortical thickness, and therefore bone strength [23]. In addition, bone development is dependent on the impact of mechanical load and processes that trigger bone modelling and remodelling [24], and possibly on structural adaptations related with trabecular microarchitecture [25].

### Sport participation and bone health

It has been shown that sport participation is crucial for healthy bone development, however not all sports have a positive influence on the skeletal mass. According to their characteristics, sports can be described as osteogenic (weight-bearing exercise) and non-osteogenic (non weight-bearing exercise). Apart from numerous health benefits [26], football is considered as an osteogenic sport both in childhood and adolescence as bone mass is augmented [27-30]. In contrast, sports such as cycling [31-40] or swimming [41-46] are associated with no change or a reduction in bone mass when compared to controls. This could be a barrier for obtaining a high peak bone mass which may compromise future bone health [40,41,46,47].

### Plyometric exercise intervention to increase bone health

To achieve the benefits of exercise and gain acceptance, PA models must be effective, simple to administer, feasible, inexpensive, short in duration and possible to perform at any location (i.e. at home, at the sports centre). Plyometric jump training (PJT) may be a judicious choice and experimental studies using animal models have repeatedly shown that short, discrete bouts of exercise interspersed with rest periods is more effective than a single longer bout of exercise for improving bone mass and strength [48].

Research in early puberty has shown that a novel and easily implemented 8-month PJT (Bounce at the Bell; ~3 min/day) enhanced bone mass at the weight bearing proximal femur [49]. Mackelvie et al. showed that a 7-month jumping intervention (10 min, 3 times/week) was associated with more bone at the FN and lumbar spine (LS) in early pubertal girls [50], and these results were maintained after 2 years [51]. In addition, prepubertal Asian and Caucasian boys of average or low body mass index (BMI) augmented bone mineral accrual at several regions after a 7-month jumping intervention (10 min, 3 times/week). However, there are a lack of studies analysing the effect of PJT in the adolescent population, which is crucial as adolescence is the period associated with the greatest increments in BMC and BMD [52]. In addition, this has not been studied in adolescents engaged in different sports (osteogenic vs. non osteogenic), which is important to examine if peak bone mass during adolescence may be maximized and therefore reduce the risk for developing osteoporosis in adulthood.

### Bone turnover markers and vitamin D

Bone development depends on its metabolic activity, which includes bone formation, resorption and, as a consequence bone turnover [53]. The relationship of PA and sport participation with bone metabolism markers has been shown previously in adolescents [54,55]. An increase in the concentrations of bone formation and resorption markers can be observed in non-osteogenic sports, such as swimming, but a comparison between osteogenic and non-osteogenic sports has not been investigated previously [56].

The role of vitamin D in bone metabolism is important due to contribution of vitamin D in calcium homeostasis and bone mineralization processes during growth. Evidence shows that adequate vitamin D levels are necessary to acquire bone mass and interact with exercise to enhance bone growth [57,58]. The magnitude of the benefits in boys and girls differ at sites of the skeleton and may depend on the baseline levels of vitamin D and on previous loading experience [59]. A positive interaction between PA and vitamin D on BMD in adolescents has been described [60,61] however the association between vitamin D with osteogenic and non-osteogenic sports has not been justified.

### Objectives

The objectives of the PRO-BONE study are: 1) to longitudinally assess, over 3 years, bone health and its metabolism in adolescents engaged in osteogenic (football) and non-osteogenic (cycling and swimming) sports, and 2) after 12 months of sport participation to examine whether a short and inexpensive 9 months PJT intervention programme is positively associated with bone-related variables and its metabolism in adolescent footballers, cyclists and swimmers.

The secondary aim of the study is to examine whether the PJT programme stimulus is enough to counteract the expected negative consequences of these non-osteogenic sports in bone health and to follow-up the bone-related variables and its metabolism over 12 months after the PJT programme.

## Methods/Design

### Study design

**Figure 1:**
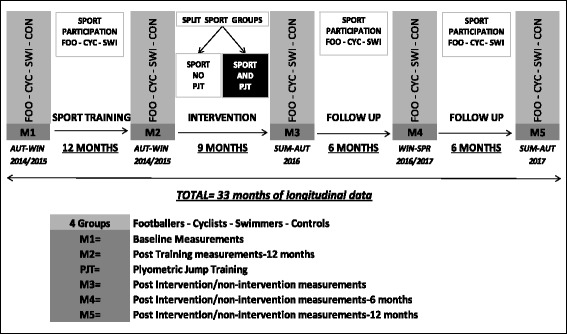
PRO-BONE timeline. FOO: Football players; CYC: Cyclists; SWI: Swimmers; PJT: Plyometric Jump Training.

PRO-BONE is a longitudinal design and involves four cohorts of males aged 12–14 years at the beginning of the study. These four cohorts consist of footballers, cyclists, swimmers and controls that will be followed over a period of 33 months. The timeline of the PRO-BONE study can be seen in Figure 1.

### Sample size

The sample size has been calculated according to the primary interest variable, TB BMD (of cyclists (aged 15.5 years) [39] in order to achieve 90% of statistical power to detect differences in the contrast of the null hypothesis H0: μ1 = μ2 through bilateral student t, difference between two dependent means (matched pairs). Taking into account a significance level of 5% and assuming that the mean of the reference group 1.133 units (SD = 0.127) and the mean of the experimental group is 1.002 units (SD = 0.093), it will be necessary to include 9 participants in the reference group and 9 participants in the experimental group, totalling 18 participants. It is known that the number of participants to recruit depends also on potential withdrawals [or could use drop-outs]: n’ = n/(1-p), so that if the withdrawals were 40% the number of participants to be recruited would be n’ = 9/(1–0.4) = 15 in each group (e.g. 15 INT cyclists + 15 CON cyclists = 30 cyclists). Therefore, cyclists (n = 30), footballers (n = 30), swimmers (n = 30) and controls (n = 15) will be recruited, yielding a total N = 105.

### Recruitment of the participants

Participants and parents/guardians will be contacted via advert flyers, posters and social media to participate in this study and by contacting sport clubs and schools from the South West of England. Where possible, a meeting will be held to explain the project as well as to answer any questions. At the end of this meeting, consent/assent forms and information sheets will be given out and participants and parents/guardians will have 15 days to return the consent/assent forms. After these 15 days, a reminder (phone call or email) will be provided to those not sending the consent/assent to check if they wish to participate. Seven more days will be given to those that agreed to participate and in the 2^nd^ reminder, they will be asked to send the interest and consent/assent forms signed.

Participants will be screened for eligibility, based on the inclusion/exclusion criteria outlined below, by a member of the research team depending on the information provided in the interest form. If eligible, the baseline assessment will be scheduled for the participant. All participants and parents involved in this project will be carefully informed about the risks and benefits of the study and will be required to sign the approved assent and consent forms before their visit to the laboratory at the Children’s Health and Exercise Research Centre (CHERC, University of Exeter).

### Inclusion and exclusion criteria

Inclusion criteria include: 1) Males 12–14 years old, engaged (≥3 h/week) in osteogenic (football) and/or non-osteogenic (swimming and cycling) sports in the last 3 years or more; 2) Male adolescents 12–14 years old not engaged in any of these sports (≥3 h/week) in the last 3 or more years (control group).

Exclusion criteria include: 1) participation in another clinical trial; 2) any acute infection lasting until < 1 week before inclusion; 3) medical history of diseases or medications affecting bone metabolism or the presence of an injury (before inclusion) that may affect participation in their respective sports and/or any variable considered in the present study (i.e. doing the PJT); 4) non-Caucasian participants. The latter is included since there are differences in body composition (bone, fat and fat-free mass) and biochemical markers (i.e. osteocalcin) between ethnic groups [62].

### Ethics approval

The methods and procedures of the PRO-BONE study have been checked and approved by: the Ethics Review Sector of Directorate-General of Research (European Commission, ref. number 618496), the Sport and Health Sciences Ethics Committee (University of Exeter, ref. number 2014/766) and the National Research Ethics Service Committee (NRES Committee South West – Cornwall & Plymouth, ref. number 14/SW/0060). All data and information obtained will be confidential and access to database will be restricted to the researchers of the study. All measurements will be carried out by qualified and experienced researchers that will undergo a Disclosure and Barring Service check for approval to work with young people.

### Study protocol and measurements

#### Body composition

##### Anthropometry

Stature (cm), seated height (cm) and body mass (kg) will be measured by using a stadiometer (Harpenden, Holtain Ltd, Crymych, UK; precision 0.1 cm; range 60–210 cm), a sitting height table (Harpenden, Holtain Ltd., Crymych, UK; precision 0.1 cm; range 32–109 cm) and an electronic scale (Seca 877, Seca Ltd, Birmingham, UK; precision 100 g; range 2–200 kg) respectively. Body mass index (BMI) will be calculated as body mass (kg) divided by the height (m) squared.

Waist circumference will measured at the midpoint between the lowest rib cage and the top of the iliac crest. Hip circumference will be measured around the widest portion of the buttocks. All measurements will be undertaken by the same trained researcher using the type Seca 201 measuring tape (Seca Ltd, Birmingham, UK; precision 0.1 cm; range 0–205 cm). All anthropometrical measurements will be performed three times and the mean will be calculated. Pubertal maturation will be self-reported by the participants during each visit using adapted drawings of the five stages (Tanner) of pubertal hair development [63].

##### Dual-energy x-ray absorptiometry

Dual-energy x-ray absorptiometry scanner (GE Lunar Healthcare Corp., Madison, WI, USA) will be used to scan participants at four sites due to the evidence of site specific impact of sports participation [64-66]: 1) LS (mean of L1-L4), 2) right hip, 3) left hip, 4) TB. The DXA equipment will be calibrated at the start of each testing day by using a LS phantom as recommended by the manufacturer. The body will be segmented in accordance to standard procedures to evaluate regional bone mass and fat distribution. The scan modes will be automatically selected by the scanner software (standard or thick). All DXA scans and analyses will be performed using the GE enCORE software (2006, version 14.10.022).

Participants will be asked to remain still and they will be scanned in the supine position. The BMC (g) and BMD (g/cm^2^) with aged-matched Z-scores and age-matched % will be obtained. For LS regions area (cm^2^), width (cm) and height (cm) will be recorded and for TB regions, fat mass (g), lean mass (kg) and body fat (% and kg) will be obtained. Information about hip strength index, fat mass ratios (trunk/total, legs/total, arms and legs/trunk), android and gynoid regions will also be obtained and have been previously validated in adolescents [67].

This technique uses ionizing radiation that raises ethical issues particularly for child participants. However, this technique uses a minimal radiation dose (similar to spending a day outside in the sunshine), and has been widely used for research purposes with child participants worldwide. The estimated lifetime risks of using GE Lunar Prodigy DXA measurements in the paediatric population was found to be negligible [68].

##### Air displacement plethysmography

Body volume will be measured with BodPod (Body Composition System, Life Measurement Instruments, Concord, California, USA) as it can effectively predict visceral adipose tissue in children [69] and determine the changes of body fat percentage over time [70]. Two measurements will be performed and if there is a difference of more than 150 mL in body volume, a third measurement will be taken. The equipment will be calibrated at the commencement of each testing day following the manufacturer’s guidelines and using a cylinder of specific volume (49.887 L). Participants will wear clothing according to the manufacturer’s recommendation (a swimsuit and a swim cap) to rule out air trapped in clothes and hair. Participants will be weighed on the BodPod calibrated digital scale and then will enter into the BodPod chamber. During the measurements participants will be asked to remain in a seated position and to breathe normally. A mean value for body volume will be obtained following the manufacturer’s recommendations [71] and this value will be integrated into the calculation of lung volume. Percentage of TB fat mass will be calculated using the equation reported by Siri [72,73].

##### Imaging bone ultrasonometer

Qualitative ultrasound measurements will be performed with a Lunar Achilles Insight and the OsteoReport PC software version 5.x + (TM Insight GE Healthcare, Milwaukee, WI, USA). This portable device measures bone stiffness using ultrasound waves and is considered a valid and radiation-free method to assess bone health in children [74,75]. The same device will be used throughout the study and calibration will be carried out prior to each visit. A standard procedure will be followed according to manufacturer’s instructions. Participants will be placed on a stable chair in a comfortable position directly in front of the Achilles device. The position of the leg will rest lightly against the calf support so the foot, calf and thigh are aligned with the centre of the calf support and the positioner.

The qualitative ultrasound device provides three outcome variables, the broadband ultrasound attenuation (BUS), the speed of sound (SOS) and the stiffness index (SI). The BUA indicates the absorption of sound waves measured in decibels per megahertz. The SOS shows the stiffness of a material by the ratio of the traversed distance to the transit time, expressed in meters per second. And the SI is calculated by a linear combination of BUA and SOS: SI = (0.67 x BUA) + (0.28 × SOS) – 420. The real-time image of the calcaneus and the region of interest ensure that the measurement is precise [74]. Both feet will be measured twice and the mean of the two measurements will be calculated and used for statistical analyses.

##### Bioelectrical impedance analysis

The portable BIA device (Tanita BF-350, Tokyo, Japan; range 2–200 kg; precision 100 g; body fat % range 1-75%; body fat% increments 0.1%) will estimate the percentage of body fat by using the values of resistance and reactance. Participants will be measured in a fasting state and will remove any metal objects and socks prior the measurements. They will be positioned on the posterior surface barefoot according to manufacturer’s instructions. Despite the reported prediction measurement error, BIA is considered a practical method to assess body fat in addition to DXA and BodPod in adolescents [76,77].

### Biochemical markers and blood collection

The measurement of bone turnover markers, in addition to the measurement of bone mass, is an interesting option to obtain a more dynamic picture of bone tissue, with the advantage that can be repeated at short intervals [78]. Therefore, the combination of both measures (bone mass and bone metabolism) is essential to obtain a better understanding on changes in the skeletal mass. In this regard, the International Osteoporosis Foundation and the International Federation of Clinical Chemistry recommended the use of serum procollagen type 1 aminoterminal propeptide (P1NP) and isomer of the Carboxi-terminal telopeptide of type 1 collagen (CTX-1) as markers for formation and resorption, respectively [79]. The role of vitamin D in bone metabolism is important due to contribution of vitamin D in calcium homeostasis and bone mineralization processes during growth. Assessment of vitamin D levels can be achieved by measuring the serum 25-hydroxyvitamin D [25(OH)D] in the blood [80]. For the scope of the present study 25(OH)D will be analysed as it has been shown to interact with PA to improve bone mass in adolescents [14].

Blood samples will be collected between 8:00 am and 9:00 am following a 12-hour fast period. A research team experienced in sampling techniques will collect capillary blood samples (~1.2 mL) from a pre-warmed hand into heparin fluoride coated microvettes (CB 300 tubes, Sarstedt Ltd, Leicester, UK) that will be placed immediately on ice. The microvettes will be centrifuged at 1000 × G per min for 15 minutes at 4°C and plasma will be separated in Eppendorf tubes of at least 60 μL, 110 μL and 60 μL and stored at −80°C for future analysis of P1NP, CTX-1 and 25(OH)D respectively. The CTX-1 and 25(OH)D biochemical markers will be analysed by using IDS-iSYS CrossLaps (Immunodiagnostic Systems Ltd, UK) and total P1NP by using ELISA kit (MyBioSource, San Diego, California, USA).

### Physical fitness assessment

A battery of tests will be used to assess attributes of physical fitness that may play an important role in the development of skeletal mass and strength during growth and maturation. Cardiorespiratory fitness (aerobic performance) will be estimated using the 20 m shuttle run test [81], which has been shown to be both reliable and valid in youth [82]. The participants will be tested at the end of the day following a standardized warm up. They will be asked to run between two lines set 20 m apart by following the pace of the audio signals produced from a CD player. The starting speed will be 8.5 km∙h^−1^ and will be increased by 0.5 km∙h^−1^ each minute. The participants will be encouraged to continue the test until they reach maximal effort. The test will end when the participant fails to reach the line two consecutive times. The last completed shuttle will indicate the score of the test.

**Figure 2:**
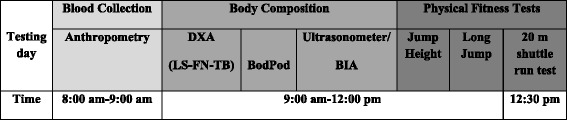
Order of the measurements for each testing day. DXA, dual energy x-ray absorptiometry; LS, lumbar spine; FN, femoral neck; TB, total body; BIA, bioelectrical impedance analysis.

The standing long jump test and the Abalakov jump test will be performed at least half an hour before the 20 m shuttle run test and following a standardized warm up and with 2 minutes rest between the two tests. The starting position of the standing long jump test will be exactly behind a line and with feet at shoulder’s width apart. Participants will be allowed to swing their arms during the eccentric contraction phase and they will be advised to jump as far as possible in order to land with both feet on a non- slippery hard surface. The distance (cm) will be measured between the starting line and the participant’s heels. Participants will perform the Abalakov jump test on a jump mat (Probotics Inc., Huntsville, USA) after having received instructions as to how much can they bend their knees and the position of their arms, they will be asked to jump as high as possible. Then, they will be placed in a standing position with their feet shoulder width apart at the jump mat. For both muscular tests the participants will perform 1 familiarization effort and 2 maximal effort jumps. The mean height and distance (in cm) of the maximal efforts will be used as criterion of measure. The reliability of both tests in adolescents was previously described and is acceptable to be used in this population [83]. The order of all the measurements for each testing day can be seen in Figure 2.

### Physical activity measurements

PA will be measured using two different methods: 1) International Physical Activity Questionnaire and 2) a wrist accelerometer (GENEActiv, GENEA, UK). The validity and reliability of the accelerometer and of the International Physical Activity Questionnaire has been established previously in children and adolescents [84,85]. GENEActiv accelerometers are waterproof so are valid for the swimmers too. Both methods will be used in order to obtain more precise data as, for example, accelerometers do not properly measure PA in cyclists as bouts of activity are not detected [86]. A diary to complement accelerometer data will be administered to the participants to obtain additional information such as calcium and protein intake.

#### Dietary assessment

Assessment of dietary intakes of calcium, vitamin D and milk will be completed by using two non-consecutive 24-h dietary recall questionnaires. CompEat Pro software (Nutrition systems, VIS Visual Information Systems Ltd., UK) will be used for the analysis.

### Jumping intervention

Following 12 months of sport specific training, the randomisation process will start in each sport group and participants will be divided into two sub-groups to perform a PJT programme as follows: 1) intervention programme groups, (sport + PJT) and 2) sport groups (sport only). It has been shown that 7 to 9 month PJT programmes can effectively improve BMC and/or BMD at different skeletal sites in children and adolescents and to maintain the benefits for 3 years after the intervention [52,87]. Therefore, a progressive PJT (~10 min/day) will be performed by intervention groups 3 to 4 times/week (depending on progression) as shown in Table 1. Before the intervention, trained staff will ensure that participants fully understand and correctly execute the different jumps and a research assistant will meet with the participants to observe, demonstrate and review the jumps. Participants will be instructed to perform a number of countermovement jumps (CMJ) and squat jumps (SJ) on a hard surface. Jumps will be performed before and after school and before going to bed. The CMJ will be performed by bending the knees prior to the jump. The CMJ activates the stretch-shortening cycle in the muscles, resulting in greater power production in the legs compared to a SJ. For the SJ participants will squat down until the knees are bent at 90 degrees, then they will immediately jump vertically as high as possible, landing back on the ground on both feet simultaneously. For this technique, the participant starts from a stationary semi-squatting position, or pauses at the lower level of the squat before jumping upwards. This removes the factor of the stretch-shortening cycle. The reliability and validity of the CMJ and SJ has been previously reported [88,89].

**Table 1:** **Plyometric jump training progression**

**Level**	**Exercise**	**Ankle weights (kg)**	**Repetitions**	^**3**^ **Sets/day (** ^**4**^ **rest)**	^**5**^ **Trainings/week**	**Jumps/week**
**1**	^1^CMJ	-	10	3	3	180
	^2^SJ	-	10	3	3	
**Total level 1 (12 weeks)**					180 x 12 =	2160
**2**	CMJ	1	10	4	3	240
	SJ	1	10	4	3	
**Total level 2 (12 weeks)**					240 x 12 =	2880
**3**	CMJ	2.5	10	4	4	320
	SJ	2.5	10	4	4	
**Total level 3 (12 weeks)**					320 x 12 =	3840
**Total intervention (36 weeks)**						8880

^1^Countermovement jump, ^2^Squat jump, ^3^1 set = 10 CMJ + 10 SJ, ^4^Rest between sets = 30 seconds.

Rest between exercises = 1 minute, ^5^When 3 sets/day, jumps will be performed in the morning before going to school (1 set), after school (1 set) and before going to bed (1 set). When 4 series, jumps will be performed in the morning before going to school (1 set), after school (2 sets) and before going to bed (1 set).

These jumps are associated with important ground reaction forces, i.e. for a countermovement it is about 5 times body weight (BW), compared to 3.5 times BW for jumping jacks. Similarly, the highest rates of change in force are 493 times BW/s for the CMJ, as shown in an independent sample of boys and girls [90]. A diary will be used to record the number of jumps performed each day. Both the intensity and number of jumps will be increased progressively in 3 levels of 12 weeks each. Intensity will be modified using ankle weights (from 1 kg at level 1 to 2.5 kg at level 3). With this an increase in BW between 2 to 5 kg will be achieved. In this regard, it has been shown that adolescents with higher BMI have higher levels of bone mass, because of the higher lean mass that they develop as a consequence of their higher fat mass [91].

## Discussion

PRO-BONE will assess the longitudinal impact of osteogenic (football) and non-osteogenic (cycling and swimming) sports on bone development in adolescents aged 12–14 years old. In addition, it will investigate whether a simple, feasible and inexpensive PJT programme can improve bone development and if the effects will be maintained a year after finishing the PJT programme. Several investigations have been conducted in order to improve bone health through exercise, strength, jumping or even combinations among them [92]. However, to achieve impact and gain acceptance, the intervention must be effective, simple to administer, feasible, inexpensive, short in duration and possible to perform at any place [49]. PRO-BONE has been designed to meet all these requirements and follow-up its effects after the withdrawal of the intervention.

Previous research has shown that exercise is positively associated with bone health [93]. However, there are some sports that due to the impact generated at the skeletal sites may have a minimal or negative effect on BMC and BMD [40,56]. As recent data have shown, jump training is associated with increases in BMC and BMD and may play an important role in the prevention of osteoporosis [94]. It is well known that early prevention is the most effective tool, therefore, it is crucial to analyse the effect of PJT at an early stage (i.e. adolescence). In this sense, it is important to examine if PJT can counteract the potential negative consequences of non-osteogenic sports on bone health and if there is enough stimuli to increase BMC and BMD in adolescents engaged in osteogenic sports.

PRO-BONE will employ different and well known technological devices and methods such as DXA, BodPod, imaging bone ultrasonometer and triaxial accelerometers among others. In addition, the PJT will include a progression in intensity with ankle weights to maximize the potential to augment bone. PRO-BONE is timely as there is a lack of studies analysing the effects of PJT on bone health during the crucial this period of life. It represents a golden opportunity to measure how a simple, feasible and inexpensive PJT is associated with bone health in adolescents engaged in different sports. It will also show if the effect of this intervention differs between sports, expecting a greater effect in cyclists and swimmers than footballers. In addition, PRO-BONE will allow us to compare within each group and investigate changes in body composition in groups doing the PJT plus training vs groups only training. Finally, PRO-BONE will examine whether PJT has any additional effect on footballers. Football is considered one of the most osteogenic sports, but this type of intervention has not yet been studied.

## Abbreviations

BMC: Bone mineral content
BMD: Bone mineral density
BMI: Body mass index
25(OH)D: 25-hydroxyvitamin D
BIA: Bioelectrical impedance analysis
BodPod: Air displacement plethysmography
BW: Body weight
SOC: Football players
SWI: Swimmers
CYC: Cyclists
CON: Control
DXA: Dual energy x-ray absorptiometry
PJT: Plyometric jump training
CMJ: Counter movement jump
SJ: Squad jump
P1NP: Procollagen type 1 aminoterminal propeptide
CTX-1: Carboxi-terminal telopeptide of type I collagen
LS: Lumbar spine
FN: Femoral neck
TB: Total body
